# Large Cervical Branchial Cleft Cyst in Adulthood Revealed by Airway Symptoms

**DOI:** 10.7759/cureus.97756

**Published:** 2025-11-25

**Authors:** Issam Berrajaa, Imane Demnati, Achraf Amine Sbai, Drissia Benfadil, Azzedine Lachker

**Affiliations:** 1 Department of Otolaryngology/Head and Neck Surgery, Mohammed VI University Hospital Centre, Oujda, MAR

**Keywords:** airway symptoms, cervical computed tomography, cervical masses, fourth branchial cleft cyst, surgery

## Abstract

Fourth branchial cleft anomalies are exceedingly rare, especially in adult patients. Their presentation can mimic other cervical masses, posing a diagnostic challenge. This case underscores the importance of considering branchial cleft cysts in the differential diagnosis of anterior neck swellings in adults.

We report a case of a 33-year-old patient who presented with a progressively enlarging anterior cervical mass associated with airway symptoms. Clinical examination revealed a soft, non-tender mass displacing the trachea. Cervical computed tomography (CT) demonstrated a well-defined cystic lesion consistent with a branchial cleft cyst. The patient underwent complete surgical excision of the mass.

Fourth branchial cleft anomalies are exceptionally rare, particularly in adults, and often present with non-specific symptoms such as dysphonia, dysphagia, or dyspnoea due to their complex anatomical course. These cysts may mimic other cervical pathologies, making their diagnosis challenging. Imaging, especially CT, plays a crucial role in identifying the lesion and its relationship to surrounding structures. Surgical excision remains the definitive treatment, with careful dissection required to preserve adjacent neurovascular and endocrine structures. Our case emphasizes the importance of including fourth branchial cleft cysts in the differential diagnosis of anterior neck masses and illustrates successful management through complete surgical excision.

Fourth branchial cleft cysts, although rare in adults, should be included in the differential diagnosis of anterior neck masses with airway symptoms. Prompt diagnosis and surgical excision offer excellent outcomes and prevent complications.

## Introduction

Branchial cleft anomalies are the second most common cause of congenital cervical tumours in children, accounting for approximately 20% of diagnosed cervical masses [1,2]. Although these malformations are relatively common, 95% of cases result from second branchial cleft lesions, while anomalies affecting the third and fourth clefts remain rare [3,4]. These malformations typically present as cervical masses, often with an inflammatory component, which can make their initial diagnosis more challenging, especially in adults. Fourth cleft cysts, in particular, due to their deep course through the neck, can manifest with compressive symptoms such as dysphonia, dysphagia, or stridor, and are frequently misdiagnosed as recurrent deep neck abscesses or thyroiditis [5].

In this report, we describe the case of a 33-year-old adult patient with an anterior cervical mass causing airway compression, diagnosed as a large fourth branchial cleft cyst. Due to the rarity of these anomalies, they are often unrecognized in clinical practice, particularly in adults, making this case especially noteworthy.

## Case presentation

A 33-year-old patient was admitted to the emergency department in April 2024 for acute respiratory discomfort associated with a left anterolateral cervical swelling. His medical history was significant for intermittent dysphonia dating back to the age of 10, with episodes recurring every one to two years. Given the protracted nature of his symptoms over a decade, the patient's recall was understandably limited to the chronology and frequency of these intermittent episodes. Upon admission, the patient was conscious and hemodynamically stable (blood pressure: 140/86 mmHg, heart rate: 87 bpm), with an oxygen saturation of 94% on room air and a respiratory rate of 22 breaths per minute.

Cervical examination revealed a left anterolateral mass, soft in consistency, measuring approximately 7 cm in its longest dimension, slightly tender to palpation, and associated with local inflammatory signs. The mass extended from the upper pole of the left thyroid lobe to the superior border of the clavicle, displacing the trachea medially and contacting the sternocleidomastoid muscle laterally. No palpable abnormalities were found in the thyroid compartment. Functionally, the patient reported paroxysmal dry cough, dysphagia to solids, and dysphonia.This voluminous mass (7 cm), by compressing adjacent structures, directly explained the patient's symptomatic triad: tracheal displacement causing dry cough, esophageal compression causing dysphagia, and laryngeal/recurrent nerve involvement causing dysphonia.

Pharyngolaryngoscopy revealed hypomobility of the left vocal cord, with no evidence of a fistulous tract originating from the piriform sinus.

Blood tests showed leukocytosis at 13,000/mm³ (normal range: 4,000-11,000/mm³), predominantly neutrophilic (9,000/mm³; normal range: 2,000-7,500/mm³), and an elevated C-reactive protein (CRP) level at 95 mg/L (normal value: <5 mg/L). Thyroid function tests were within normal limits.

**Figure 1:**
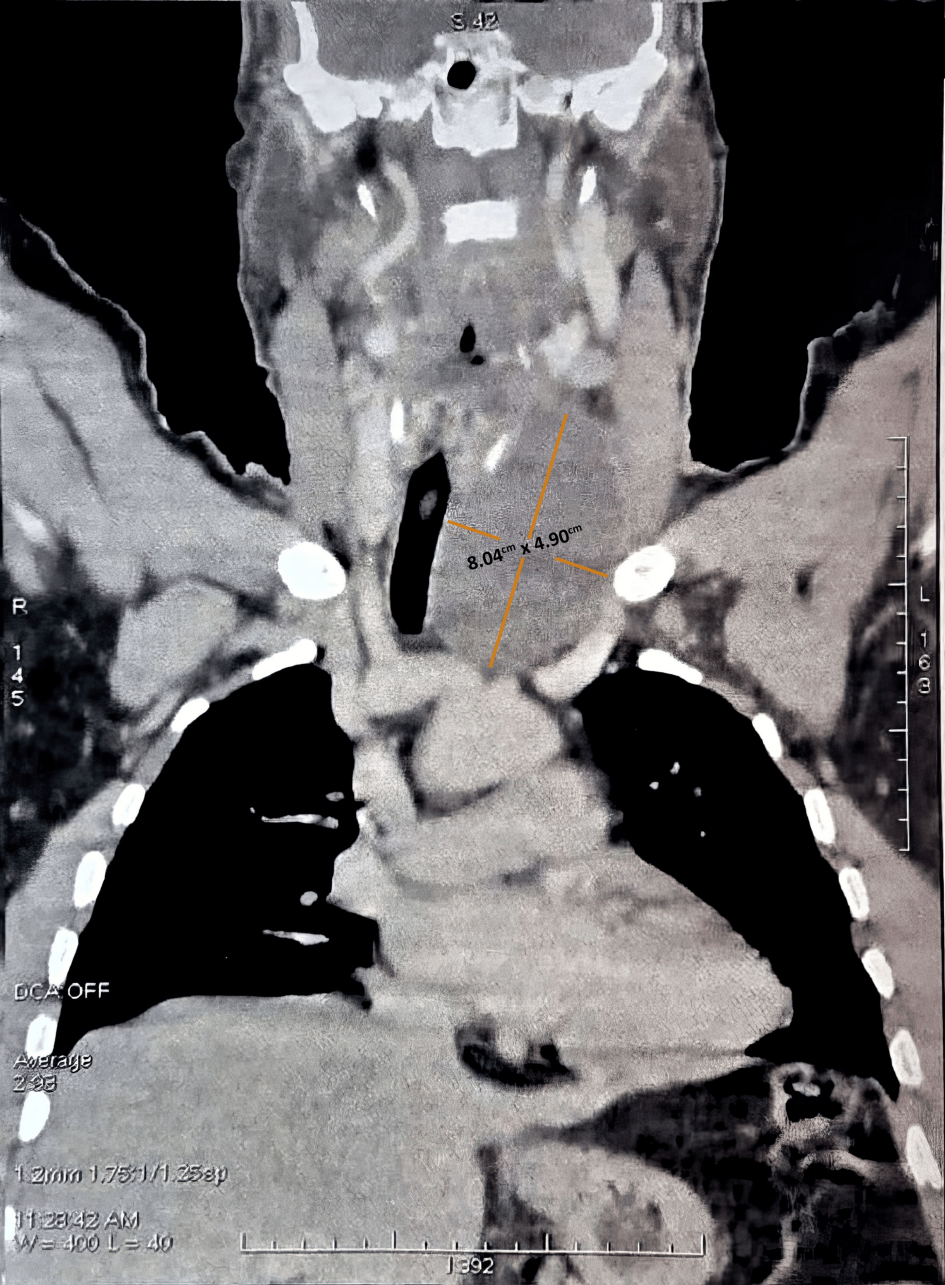
Coronal CT scan of the neck and upper mediastinum showing a large left paratracheal cystic mass.

A cervical computed tomography (CT) scan was performed, revealing a well-defined, left paratracheal cystic mass with thin regular walls and hypodense content, enhancing after contrast injection. The lesion measured 8.04 cm in length by 4.90 cm in width on axial sections. It displaced the trachea to the right without compression, pushed the thyroid gland anteriorly, and was in close contact with the left thyroid lobe (Figure 1).

The patient received broad-spectrum antibiotic therapy (amoxicillin-clavulanic acid 1 g every eight hours for 10 days). At the end of the antibiotic course, follow-up clinical examination showed regression of local inflammatory signs. However, due to the persistence of the swelling, surgical intervention was indicated.

**Figure 2:**
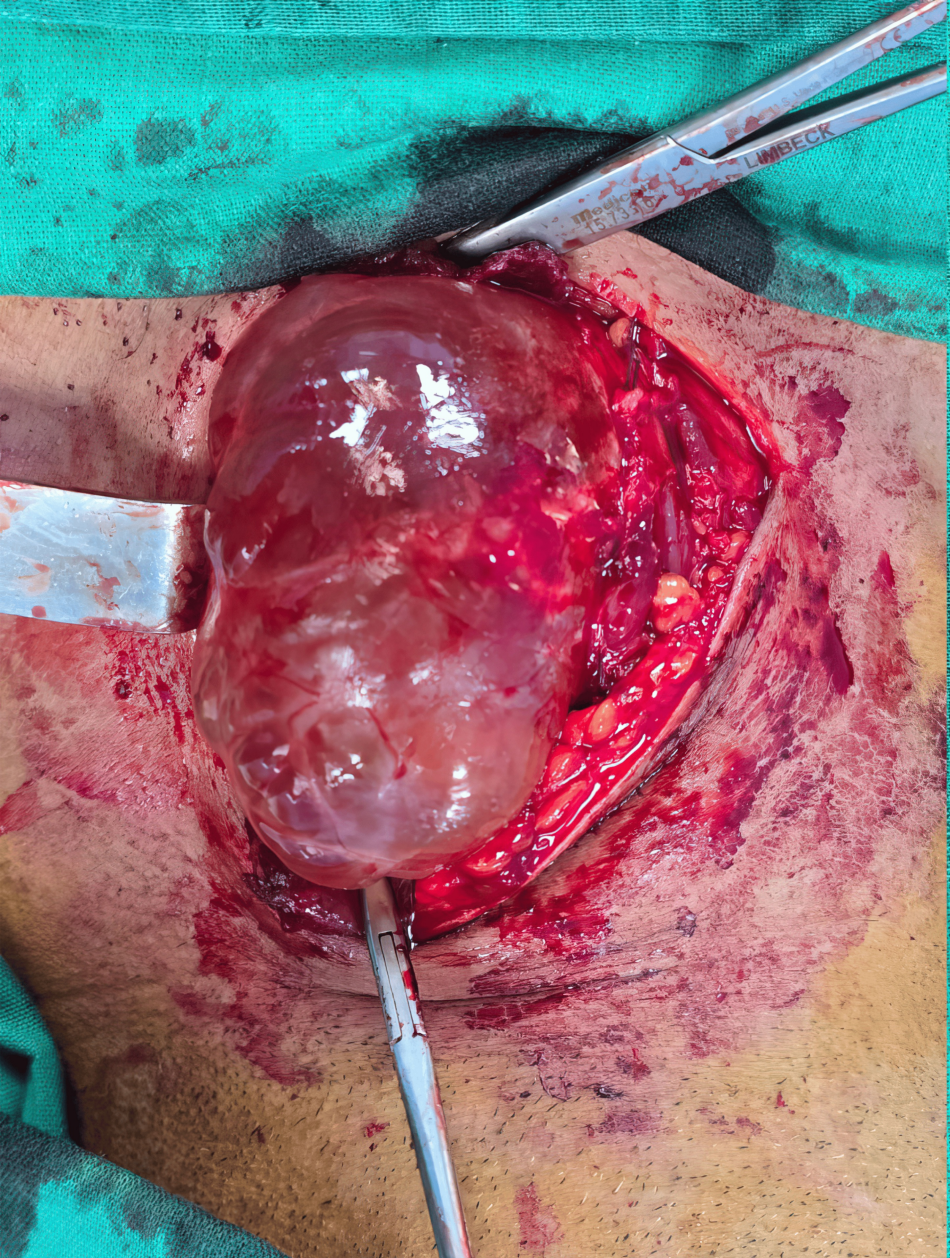
Intraoperative view showing the cyst during its excision.

**Figure 3:**
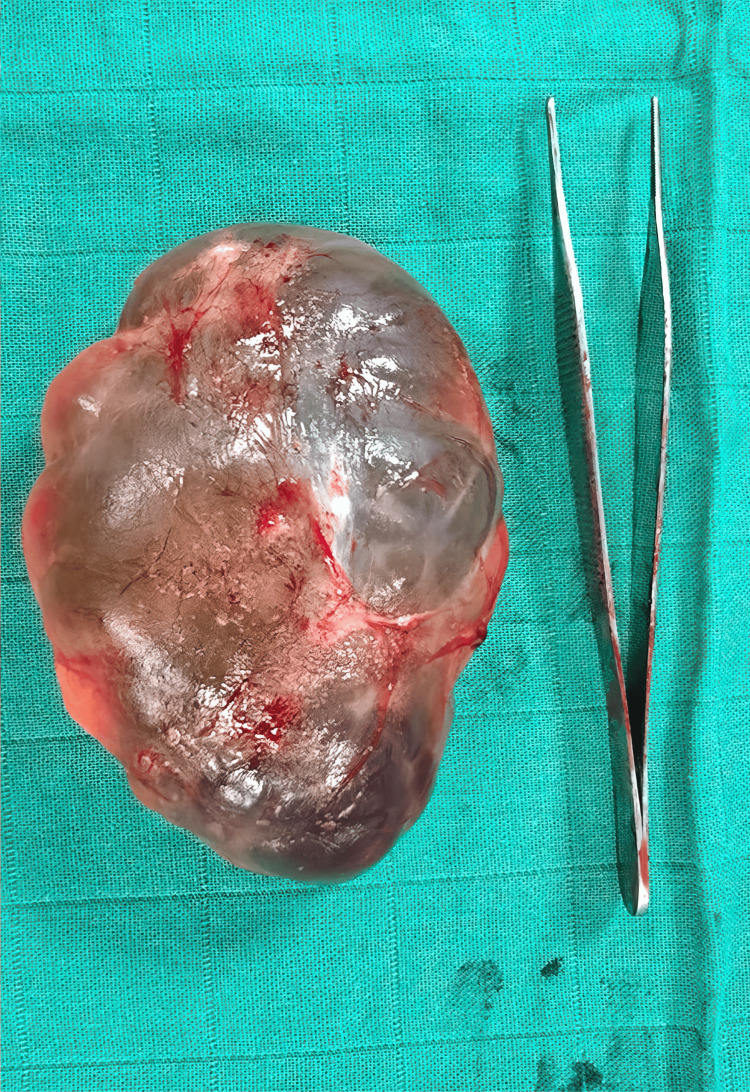
Macroscopic view of the excised cyst specimen.

A transverse incision was made along a natural skin crease on the left side of the neck (Figure 2), providing direct access to the cystic lesion. The left superior parathyroid gland was identified and carefully preserved during dissection. The left recurrent laryngeal nerve was also visualized and kept intact. The cystic mass, well-defined and encapsulated (Figure 3), had displaced the left thyroid lobe. The thyroid parenchyma of the left lobe was adherent to the cyst wall.

Following a complete excision of the mass, the thyroid capsule appeared thickened and reddish, with whitish areas. A left thyroid lobectomy was therefore performed. No sinus tract was identified. Postoperative recovery was uneventful, and the patient was discharged on postoperative day two.

**Figure 4:**
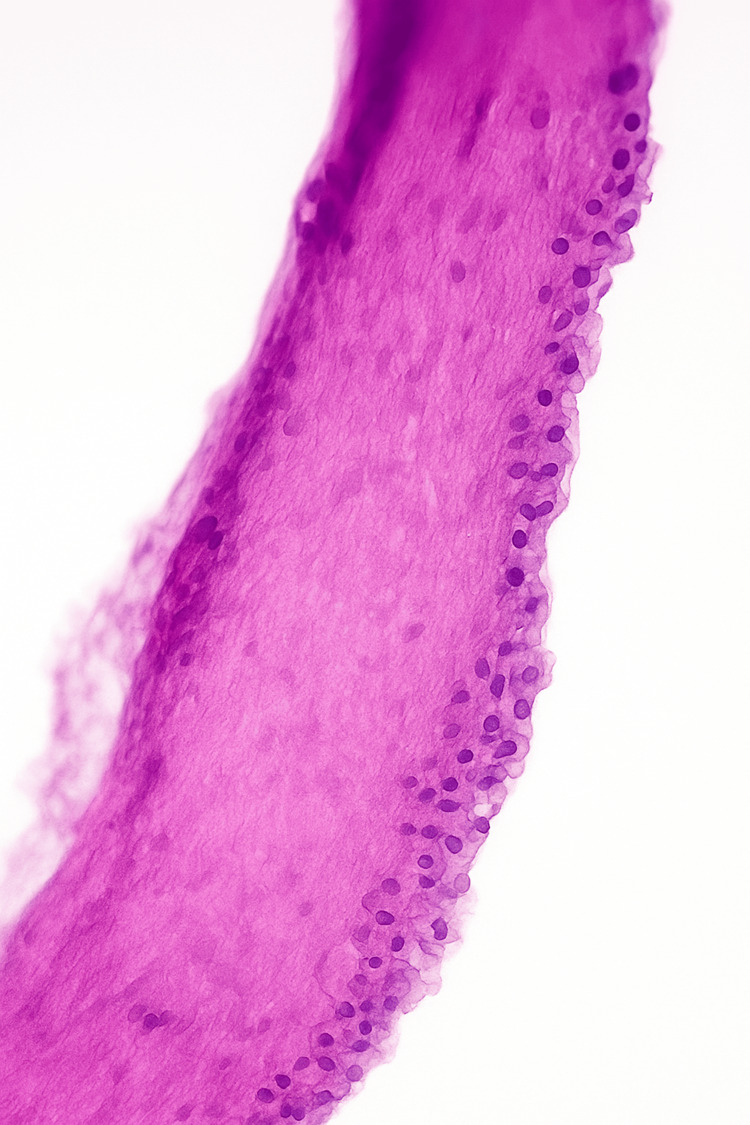
Cyst wall lined by ciliated pseudostratified epithelium with lymphoid aggregates and chronic inflammation, consistent with a branchial cleft cyst (Hematoxylin and Eosin stain, H&E; original magnification ×200).

Microscopic examination of the specimen revealed a cystic cavity lined predominantly by stratified squamous non-keratinized epithelium, with focal areas lined by ciliated columnar epithelium (Figure 4). The cyst wall consisted of fibrous connective tissue containing multiple well-developed lymphoid follicles with prominent germinal centers. Focal areas of chronic inflammatory cell infiltration were noted, composed mainly of lymphocytes and plasma cells.

The thyroid capsule adjacent to the lesion showed fibrous thickening and patchy hyalinization, with scattered zones of congestion. No malignant cells or granulomatous changes were observed. Special staining with hematoxylin and eosin (H&E) confirmed the above features.

These histopathological characteristics, in conjunction with the lesion’s deep paratracheal location, close relationship to the upper pole of the thyroid lobe, and absence of a sinus tract to the skin, support the diagnosis of a fourth branchial cleft cyst.

The patient's postoperative course was uneventful, and he was discharged from the hospital on the third postoperative day.

Clinical and paraclinical follow-up was conducted for 18 months. During this period, the patient reported a complete and lasting resolution of his preoperative symptoms, including dysphagia, dysphonia, and respiratory distress. Clinical examination confirmed a well-healed surgical scar, without any palpable mass or signs of recurrence. Additional tests, including thyroid function tests, remained normal throughout this follow-up period.

## Discussion

The branchial apparatus is composed of six pairs of mesodermal arches, separated by endodermal invaginations on the inside and ectodermal invaginations on the outside, respectively called pharyngeal pouches and branchial clefts. The majority of branchial anomalies, which present as fistulas, sinuses, or cysts, arise from the remnants of the first and second branchial clefts, accounting for up to 90% of branchial anomalies. In contrast, anomalies derived from the third and fourth branchial clefts are extremely rare [6,7].

The diagnosis of fourth branchial cleft cysts is particularly challenging due to their rarity and non-specific presentation. The differential diagnosis for a cervical mass with inflammatory signs in an adult includes several more common entities. These primarily encompass infectious processes such as deep neck abscesses and suppurative lymphadenitis, congenital lesions like second or third branchial cleft cysts, thyroglossal duct cysts, and lymphatic malformations. Neoplastic causes must also be considered, including cystic metastatic lymph nodes (particularly from papillary thyroid carcinoma or oropharyngeal cancers), thymic cysts, and soft tissue tumors. The initial clinical presentation often overlaps significantly with these conditions, making fourth branchial cleft cysts a diagnosis of exclusion in many cases.

From an epidemiological perspective, first branchial cleft anomalies account for 5%-25% of cases, second cleft anomalies are the most common (40%-95%), third cleft anomalies are rare (2%-8%), and fourth cleft anomalies represent less than 1% of all branchial cleft anomalies [8]. Anomalies of the third and fourth branchial clefts can present with similar clinical features, making differentiation essential. Third branchial cleft tracts typically arise from the lower anterior neck, pass posterior to the internal carotid artery, course between the glossopharyngeal and hypoglossal nerves, and connect to the pyriform sinus - often superior to the superior laryngeal nerve [9]. In contrast, fourth branchial cleft tracts are more frequently left-sided, pass deep to the common carotid artery, loop around the aortic arch (on the left) or the subclavian artery (on the right), and then ascend superficial to the recurrent laryngeal and hypoglossal nerves before terminating in the upper pole of the thyroid lobe [10-12]. Anomalies of the fourth branchial arch most often present as a sinus extending from the apex of the piriform fossa to the upper lobe of the left thyroid. These result from an embryonic remnant originating at the piriform sinus and descending through the piriform fossa into the mediastinum along the tracheoesophageal groove, terminating in the neck, behind the internal and common carotid arteries. A fourth branchial cleft cyst is distinguished from other branchial anomalies by its specific anatomical location: it is laterally bordered by the sternocleidomastoid muscle, medially by the trachea, anteriorly by the common carotid artery, and anteromedially by the infrahyoid muscles [13,14].

This anatomical pathway explains the diverse locations of fourth branchial cleft cysts, which can be found in the thyroid gland, the neck, and the mediastinum. It also accounts for the symptoms presented by our patient, including (i) *dysphonia*: likely caused by inflammation or irritation of the recurrent laryngeal nerve due to the cyst's proximity. In some cases, prolonged compression can lead to transient vocal cord paralysis; (ii) *selective dysphagia to solids*: resulting from narrowing of the cervical esophagus caused by inflammation and compression from the cystic mass; and (iii) *dyspnea*: due to tracheal deviation observed on imaging, which, in extreme cases, may progress to respiratory obstruction requiring urgent intervention.

A structured, stepwise approach combining detailed clinical history, comprehensive physical examination, and targeted complementary investigations - including high-resolution imaging (ultrasound, CT, MRI), endoscopic evaluation of the upper aerodigestive tract (direct laryngoscopy, hypopharyngoscopy), and, when indicated, functional studies such as barium swallow. This approach aims to precisely characterize the lesion, identify its anatomical relationships, detect any associated sinus tract or fistulous communication, and rule out other potential causes of cervical masses, particularly in adults where atypical presentations are more frequent.

Imaging plays a pivotal role in diagnosing piriform sinus anomalies, particularly fourth branchial cleft cysts. CT and MRI are essential for delineating the lesion, defining its anatomical relationships, and assessing its extension. Several studies have described characteristic radiologic features that help distinguish branchial cleft cysts from other cervical lesions [15]. CT scan is considered the imaging modality of choice for evaluating the location and extent of piriform sinus anomalies. It is superior to MRI in detecting air within the sinus tract and identifying inflammatory changes involving the thyroid gland, strap muscles, and surrounding tissues [16]. However, the visualization of the internal opening of the sinus tract can be hindered by inflammation or edema, potentially reducing CT’s diagnostic sensitivity.

In such cases, a barium esophagogram can serve as a useful complementary investigation. Nevertheless, its diagnostic yield is variable. One report showed that barium swallow studies detected piriform sinus fistulas in only 50% of cases that were later confirmed by direct laryngoscopy [17]. The timing of the study is critical; performing the esophagogram too soon after abscess drainage may lead to false-negative results. Some authors suggest that performing a CT scan immediately following barium ingestion can enhance the tract’s visibility by improving local contrast [18]. Unlike Lin and Wang, who showed that the sinus tract was identified by barium scans in almost all cases [19], highlighting the importance of optimized imaging timing and technique, some studies report a much lower sensitivity, particularly in cases with recent infection or inflammation obscuring the sinus opening.

Following the resolution of the infection, the standard treatment is complete surgical excision of the cyst, sinus tract, and piriform attachment [20]. In certain cases, partial thyroid lobectomy may be required to ensure complete resection [21]. Given the anatomical complexity of these anomalies, preserving adjacent structures, particularly the recurrent laryngeal nerve, thyroid gland, and parathyroid glands, is crucial. In our case, the left thyroid lobe was displaced anteriorly, with an inflamed parenchyma and whitish deposits, associated with firm adhesions to the cyst wall. These anatomical changes justified performing a left thyroid lobectomy despite the absence of visible suppuration on preoperative imaging. Thyroid function was preserved both before and after surgery. Other therapeutic approaches have been described in the literature, including minimally invasive endoscopic cauterization, which is an alternative for small lesions, particularly in younger patients, and sclerotherapy, although its effectiveness remains limited for larger masses.

However, for large or recurrent lesions, surgical excision remains the most effective treatment option. In our case, complete excision of the cyst was successfully performed, and the postoperative follow-up revealed no complications. This case underscores the rarity of fourth branchial cleft cysts diagnosed in adulthood, highlighting the need for a multidisciplinary approach integrating imaging, endoscopy, and surgery to optimize both diagnosis and treatment.

To provide further clinical context and highlight the rarity of our case, we conducted a comparative analysis of published adult cases of fourth branchial cleft cysts. This review focuses on key parameters such as patient age, presenting symptoms, cyst dimensions, and therapeutic strategies (Table 1).

**Table 1:** Comparative summary of published adult cases of fourth branchial cleft cysts Note: The comparative data presented in this table were extracted from the cited references [11,12,18].

Author (Year)	Age (years)	Symptoms	Cyst Size (cm)	Treatment
Pereira et al. (2004) [18]	16	Pain and swelling in the left side of the neck	Not reported	Surgical excision+hemithyroidectomy
Meng et al. (2018) [11]	63	Cervical mass with mediastinal extension since childhood	12×12	Surgical excision without a partial thyroidectomy
Li et al. (2023) [12]	44	Posterior mediastinal mass	3.06x2.51	Thoracic surgery
Present case (2025)	33	Cervical mass with mediastinal extension+dysphonia	8.04x4.90	Excision+left lobectomy

To contextualize the clinical and surgical particularities of our case, we compared it to previously published adult cases of fourth branchial cleft cysts (Table 1). Only a limited number of reports described cysts of such considerable size. Our case stands out due to the large dimensions of the lesion (8.04×4.90 cm), the associated vocal cord dysfunction, and the requirement for left lobectomy due to significant inflammatory adhesions.

## Conclusions

The diagnosis and management of a fourth branchial cleft cyst in an adult patient remain clinical challenges due to the rarity of this condition at this age. Although these cysts are typically diagnosed during childhood, their discovery in adults may be delayed, often due to nonspecific symptoms or asymptomatic progression.

Our case also highlights the need for heightened vigilance when evaluating branchial anomalies in adults, considering the anatomical complexity and potential variations. Long-term follow-up remains essential to monitor for possible thyroid abnormalities or other complications associated with this rare condition.
